# Prevalence and Predictors of Post-Traumatic Stress Disorder Among Detainees During the Sudanese Period of Political Instability in Khartoum State (2018–2019)

**DOI:** 10.1192/bjo.2026.11069

**Published:** 2026-06-30

**Authors:** Ruba Mohamed, Reem Ibrahim, Reem Abdulghani, Syed Fahad Javaid, Mohamed Alnor

**Affiliations:** 1 Faculty of Medicine, Sudan International University, Khartoum, Sudan; 2 Department of Psychiatry, College of Medicine and Health Sciences, United Arab Emirates University, Al Ain, UAE; 3 Behavioral Science Institute, Al Ain Hospital, Al Ain, UAE

## Abstract

**Aims:**

Political violence, detention and torture cause severe psychological morbidity. During a period of political instability in Sudan between December 2018 and June 2019, civilians were exposed to extreme cumulative trauma, yet data on post-traumatic stress disorder (PTSD) in this population remain limited. This study aimed to estimate the prevalence of PTSD among detainees, examine symptom characteristics and psychometric properties of the PTSD scale, and identify predictors of PTSD in a conflict-affected population.

**Methods:**

A cross-sectional study was conducted among 246 Sudanese individuals who experienced detention, torture or related trauma in Khartoum State between December 2018 and June 2019. PTSD symptoms were assessed using DSM–IV criteria, including functional impairment. Demographic characteristics and trauma exposure variables (type, number and timing of events) were examined. Psychometric analyses included internal consistency testing and receiver operating characteristic (ROC) analysis to determine a screening cut-off. Multivariate logistic regression with stepwise selection was used to identify predictors of probable PTSD.

**Results:**

Participants were predominantly male (89.8%), aged 20–29 years (72.0%) and students (54.1%). Trauma exposure was extensive and cumulative: 45.5% reported exposure to all four trauma categories, and 87.2% of torture survivors experienced multiple torture methods. PTSD prevalence was high, with 65.9% meeting symptom criteria and 38.2% meeting diagnostic criteria including functional impairment. The most commonly reportedsymptoms were irritability (77.8%), re-experiencing symptoms (71.1%) and concentration difficulties (67.9%). The PTSD scale showed good internal consistency (Cronbach’s α=0.82). ROC analysis identified a threshold of ≥7 symptoms as the optimal screening cut-off (area under the curve 0.84). In multivariate analysis, exposure during the most intense period of violence in June 2019 (3–30 June) was the strongest PTSD predictor (adjusted odds ratio 4.99), followed by witnessing trauma, exposure to shocking events involving family or friends, intense emotional response and direct exposure to beating or torture. A clear dose–response relationship was observed between cumulative trauma exposure and PTSD severity.

**Conclusion:**

PTSD prevalence among detainees during this period of political instability in Sudan was alarmingly high, particularly among those exposed during peak violence. Temporal context, indirect trauma involving loved ones and cumulative exposure were stronger predictors than demographic factors. These findings highlight the urgent need for trauma-informed mental health services, screening and psychosocial support for survivors of political violence in Sudan and similar conflict-affected settings.

